# The role of natriuretic peptides in volume assessment and mortality prediction in Haemodialysis patients

**DOI:** 10.1186/s12882-015-0212-4

**Published:** 2015-12-29

**Authors:** Murugan Sivalingam, Enric Vilar, Suresh Mathavakkannan, Ken Farrington

**Affiliations:** Renal Unit, Lister Hospital, Stevenage, Herts SG1 4AB UK

## Abstract

**Background:**

Maintaining optimal fluid balance is essential in haemodialysis (HD) patients but clinical evaluation remains problematic. Other technologies such as bioimpedance are emerging as valuable adjuncts. This study was undertaken to explore the potential utility of the natriuretic peptides – atrial natriuretic peptide (ANP) and B-type natriuretic peptide (BNP) in the assessment of fluid status and cardiovascular risk in this setting.

**Methods:**

This was a cross-sectional study carried out in an unselected cohort of 170 prevalent HD patients. Volume status was assessed by clinical parameters – the presence or absence of peripheral oedema, raised jugular venous pressure and basal lung crepitations; by extracellular fluid volume (ECFV) status determined by whole body bioimpedance; and by serum levels of BNP and ANP (pre- and post –dialysis). The relationships of ANP and BNP levels to clinical and bioimpedance parameters of volume status was determined. Patients were followed up for 5 years to assess the relationship of natriuretic peptide levels to mortality.

**Results:**

Bioimpedance estimates of ECFV expansion (>105 % of ideal ECFV) was present in 52 % of patients pre-dialysis. A significant proportion (21 %) of pre-dialysis patients had a depleted ECFV (<95 % of ideal ECFV) pre-dialysis. The situation was reversed post-dialysis. A raised JVP >3 cm was the most reliable clinical sign of ECFV expansion inferred from bioimpedance measurements and natriuretic peptide levels. The vast majority of patients with this sign also had lung crepitations or peripheral oedema or both. BNP was a stronger predictor of ECFV expansion than either pre- or post-dialysis ANP. BNP was also a stronger predictor of five-year survival.

**Conclusion:**

Serum levels of BNP have a strong relationship to both volume status and survival in HD patients. We found no clear role for measurement of ANP, though changes in blood levels may be a sensitive indicator of acute changes in volume status. Whether monitoring levels of these peptides has a role in the management of volume status and cardiovascular risk requires further study.

## Background

The maintenance of optimum fluid balance in patients on haemodialysis (HD) is a key therapeutic goal, and plays a major role in determining morbidity and mortality. Clinical assessment remains the main arbiter of volume status though relatively insensitive. Other measures may be helpful, the most well explored of these being bioimpedance methodologies. Blood levels of a biomarker of fluid status would be of huge benefit. Natriuretic peptide are candidate biomarkers which have shown promise in other settings, but their role in this setting is not well defined. A number of studies have compared the performance of plasma B-type Natriuretic Peptide (BNP) levels, clinical assessment, and bioimpedance methods [1–4] None to our knowledge have included Atrial Natriuretic Peptide (ANP) in these comparisons.

Natriuretic peptides play a major role in salt and water homeostasis, protecting the cardiovascular system from the effects of volume overload. ANP (28 amino acids) and BNP (32 amino acids) share a common 17-amino-acid ring structure. Both peptides are released primarily from the heart and act in various tissues inducing vasodilatation, natriuresis and diuresis [5]. ANP is predominantly synthesised in the atria and BNP in the ventricles, though both can be synthesised in either chamber under pathological conditions [6]. ANP is stored in atrial granules and released even with minor increases in volume whilst BNP, which only has minimal storage in granules, is synthesised and secreted in bursts [7]. ANP is stored as pre-proANP, which is then cleaved to proANP and then finally to the inactive NT proANP (1–98) and the biologically active ANP (99–126). Synthesis of pre-proBNP in the ventricle is initiated by wall stress due to either volume expansion or pressure overload [8]. Pre-proBNP is cleaved initially to proBNP (1–108) and then to the biologically active BNP (1–32) and the inactive amino-terminal fragment (NT proBNP). The biological actions of natriuretic peptides are mediated by binding with specific membrane bound receptors and activation of the guanyl cyclase system [9]. Both peptides are inactivated by enzymatic degradation by neutral endopeptidase and lysosomal degradation after uptake by the clearance receptor [10]. ANP binds to the clearance receptor with greater affinity than BNP and so has a shorter half-life. The half-life of ANP is 2–3 min whilst that of BNP is around ten-fold greater [11].

Both ANP and BNP have been investigated as markers of hydration in dialysis patients. Mean ANP levels are markedly increased in dialysis patients, decrease with ultrafiltration, but remain constant during isovolaemic HD suggesting that reduced synthesis in response to decreasing circulating volume is their main determinant [12]. Elevated ANP levels post-dialysis are associated with fluid overload. Consistent weight reduction was followed by a decrease of ANP levels but the levels remained high compared with levels normal subjects. Other studies have reported similar findings [13–17].

Blood levels of BNP also reflect volume status in dialysis patients [4, 18], in addition they are strongly associated with left ventricular dysfunction [19]. There is data though to suggest that NT proBNP levels may be more reflective of volume overload than of cardiac dysfunction [20]. BNP has been shown to be more closely correlated with left ventricular mass index and ejection fraction than ANP and in this study only BNP was an independent predictor of death [21]. BNP levels decrease significantly during high-flux HD, dialyser clearance being the major contributant [22, 23]. Volume change appears to play a much smaller role – suggesting a relative insensitivity of BNP secretion to acute volume change [22, 23]. In keeping with this is the lack of change of BNP levels during low-flux HD in spite of a reduction in volume status due to ultrafiltration [18]. In the same study ANP levels fell significantly during the dialysis session, suggesting ANP to be a better marker of acute volume change than BNP

In summary comparative studies of ANP and BNP have suggested that in this setting ANP may be the better marker of volume status and BNP might the better marker of cardiac dysfunction. Hence we sought to assess the clinical utility of pre-dialysis ANP levels and changes in these levels during a dialysis session as markers of volume status assessed clinically and by bioimpedance. Predialysis BNP levels were taken, primarily as an indication of cardiac functional status. We also aimed to investigate the association between these biomarkers and survival.

## Methods

### Study population

Patients undergoing outpatient HD under the auspices of the Lister Renal Unit, Stevenage UK were supplied with details of the study and subsequently approached to take part. There were no exclusion criteria except the inability to give informed consent. A non-selective recruitment strategy was pursued in order to ensure that the findings of the study were applicable to a typical dialysis population. The study was approved by the Hertfordshire Ethical Review Committee.

### Haemodialysis programme

All patients received thrice-weekly HD using high-flux membranes, predominantly polysulphone. Water quality was regularly monitored to ensured tight bacteriological standards [<0.1 cfl/ml and <0.03 EU/ml]. Around 50 % of patients were treated by on-line post-dilution haemodiafiltration (HDF). Dialysis was individualised and prescribed and monitored according to a target two-pool total *Kt*/*V* of 1.2 per session, composed of dialysis component (*Kt*/*V*_dialysis_) plus a component derived from residual renal urea clearance (*Kt*/*V*_renal_). To achieve this pre- and post-dialysis blood urea levels and interdialytic urine collections were carried out monthly. Dialysis fluid contained sodium (138 mmol/L); potassium (2 mmol/L); calcium (1.25 mmol/L); magnesium (0.5 mmol/L); chloride (108.5 mmol/L); bicarbonate (35 mmol/L) and glucose (5.5 mmol/L). Dialysate temperature was 36 °C.

### Protocol

1. Informed written consent obtained.
2. Patients were studied during a single dialysis session in mid-2008.
3. Predialysis clinical examination was carried out
4. Extracellular fluid volumes were measured using whole-body bioimpedance at the beginning of dialysis session.
5. Two samples for serum ANP was taken – one at the start (pre dialysis) and one at the end of the dialysis (post dialysis). A single sample was taken for serum BNP was also taken pre dialysis.
6. Routine demographic, clinical, biochemical and haematological data were retrieved from case-notes and electronic records
7. Patients were followed up through their electronic patient record to death on dialysis or for 5 years. Follow up was discontinued after transplantation or transfer to another dialysis centre and survival data censored at the date of transplantation or transfer.

### Clinical examination

A detailed clinical examination was carried out by one of two experienced renal registrars, to assess each patient’s volume status.

Note was made of the presence or absence of:

- an elevated JVP – measured with respect to the sternal angle
- auscultatory crackles at the lung bases
- peripheral oedema.

### Routine data collection

The following data was collected on all patients

1. Demographic parameters including age, sex, dialysis vintage
2. Clinical parameters including primary renal disease, the presence of diabetes, and the presence of cardiac disease and other comorbidities. The presence of cardiac disease was inferred by a history of Myocardial Infarction, invasive coronary intervention, angina, chronic atrial fibrillation, cardiomyopathy, or valvular heart disease. A record of anti-hypertensive drug therapy was recorded The Charlson Comorbidity Index (CCI) was also calculated [24].
3. Details of dialysis session, including pre- and post-dialysis body weight and blood pressure, ultrafiltration volume (UFV) and ultrafiltration rate (UFR), and duration of dialysis session (Td).
4. Data relating to the immediately preceding monthly monitoring round including sessional Kt/V, residual renal function (KRU), serum albumin, and C-reactive protein (CRP)

### Biochemical measurements

#### ANP

αANP was measured by ELIZA assay (Peninsula Laboratories, San Carlos, CA, USA). Blood samples were taken in chilled EDTA tubes, centrifuged at 5 °C, plasma separated and stored at –20C. The normal concentrations vary between 15 and 40 pg/ml.

#### BNP

BNP _77–108_ (carboxyl terminal peptide or BNP 32) was measured using a Triage BNP Assay kit (Biomed diagnostics incorporated, San Diego, CA). This kit deploys an immunofluorescence assay using a 0.5 ml EDTA blood sample with normal range <100 pg/ml.

### Whole body bioimpedance

Whole body bioimpedance measurements were carried out using the Xitron 4200. The device uses the Cole model to derive the resistance values and has the software algorithm built-in to calculate the respective fluid volumes. We derived estimates of excess ECF using the method described by Chamney [25] which utilises the slope between ECF volume (bioimpedance) and body weight at normovolaemia. For males the slope was 0.239 L/kg and for females 0.214 L/kg. Using these values “ideal ECF” was determined and Excess ECF as the difference between measured ECF and “ideal ECF”.

### Statistical analysis

All data with a normal distribution are presented as mean ± sd. Non-normally distributed data is presented as median (Interquartile range). The distributions of ANP, BNP, KRU and CRP values were not normal. The Students “t” test, ANOVA, the Mann–Whitney test, and the Kruskal-Wallis test were used as appropriate to analyse the differences between the groups. A p-value of <0.05 was assumed to indicate statistical significance. The diagnostic utility of ANP and BNP in predicting volume overload and survival was analysed using the Receiver Operating Characteristics curve (ROC) analysis. Area under the curve (AUC) was compared for each of the peptide measurements. The predictors of volume overload (defined as ECFV >105 % of ideal ECF as determined by bioimpedance [25]) were determined in stepped logistic regression analysis (Backwards LR). The predictors for survival were assessed using Cox Proportional Hazards regression analysis controlled for age, sex, ethnicity, pre-dialysis weight, residual urea clearance > 1 ml/min, CCI, serum albumin, and elevated CRP (>5 mg/l). Values of ANP and BNP were logarithmically transformed for use in these regression analyses. SPSS version 19 was used for all these analyses.

## Results

One hundred and seventy patients were studied (Table 1). Mean age was 66 ± 13 years. Sixty-eight percent of the patients were male. There were no significant gender differences in pre dialysis ANP and BNP but there was a trend toward post dialysis ANP being higher in men compared to women (86.8 [IQR 116] vs. 64.5 [IQR 74] pg/ml, *p* = 0.053). Forty-four (26 %) had diabetes. Median RRT vintage was 42 [48 IQR] months. Fifty-six (33 %) had a residual urea clearance > 1 ml/min. Mean sessional Kt/V was 1.29 ± 0.21, with a mean duration (Td) of 184 ± 30 mins. Mean pre-dialysis weight was 73.7 ± 15.4 kg.

**Table 1:** Demographic, clinical, biochemical, and bioimpedance characteristics in 170 dialysis patients

Demographic and Clinical Characteristics	
Number	170
Age (Years)	66 ± 13
Gender (% Male)	68
Ethnicity (%White)	84
Predialysis weight (kg)	73.7 ± 15.4
Diabetes (%)	26
Cardiac Disease (%)	45
Cancer (%)	13
Charlson Comorbidity Index	5.7 ± 2.1
RRT Vintage (months)	42 [48]
Biochemical Parameters	
Serum Sodium (mmol/l)	136.9 ± 3.2
Serum albumin (g/l)	35.5 ± 4.6
Haemoglobin level (g/l)	11.6 ± 1.4
High CRP >5 mg/l (%)	48
KRU (ml/min)	0.3 [1.5]
KRU > 1 ml/min (%)	33
Dialysis Parameters	
Dialysis session time (minutes)	184 ± 30
Sessional Kt/V	1.29 ± 0.21
Ultrafiltration volume (litres)	1.7 [1.4]
Clinical Assessment of Volume	
Pre-dialysis Systolic BP (mmHg)	151 ± 25
Pre-dialysis Diastolic BP (mm Hg)	79 ± 15
Post-dialysis Systolic BP (mmHg)	137 ± 26
Antihypertensive medication (%)	74
Oedema (%)	37
JVP > 3 cm (%)	38
Basal Crepitations (%)	54
Bioimpedance Parameters	
ECFV (l)	17.91 ± 3.98
Ideal ECFV (Chamney)	17.09 ± 4.03
Excess ECFV	0.82 ± 2.46
Natriuretic Peptide levels	
Pre-dialysis ANP (pmol/l)	169 [208]
Post-dialysis ANP (pmol/l)	79 [106]
Pre-dialysis BNP (pmol/l)	325 [1206]

*RRT* renal replacement therapy, *CRP* C reactive protein, *KRU* residual urea clearance (ml/min). *ECFV* extracellular fluid volume, *BP* blood pressure

### Clinical signs of fluid overload

Mean ultrafiltration volume (UFV) was 1.7 [1.4 IQR] litres per session (Table 1). Mean systolic BP was 151 ± 25 (pre-dialysis) and 137 ± 26 mmHg (post-dialysis). Corresponding diastolic pressures were 79 ± 15 and 73 ± 13 mmHg respectively. Sixty-three patients (37 %) were judged as having clinical signs of oedema, 65 (38 %) as having a raised JVP (>3 cm) and as having 92 (54 %) basal crepitations on lung auscultation (Table 1). Thirty-three patients (19 %) had all three of these signs, 34 (20 %) had two, 52 (31 %) had one, and only 51 (30 %) had none.

### Extracellular fluid volume estimates by bioimpedance

**Fig. 1:**
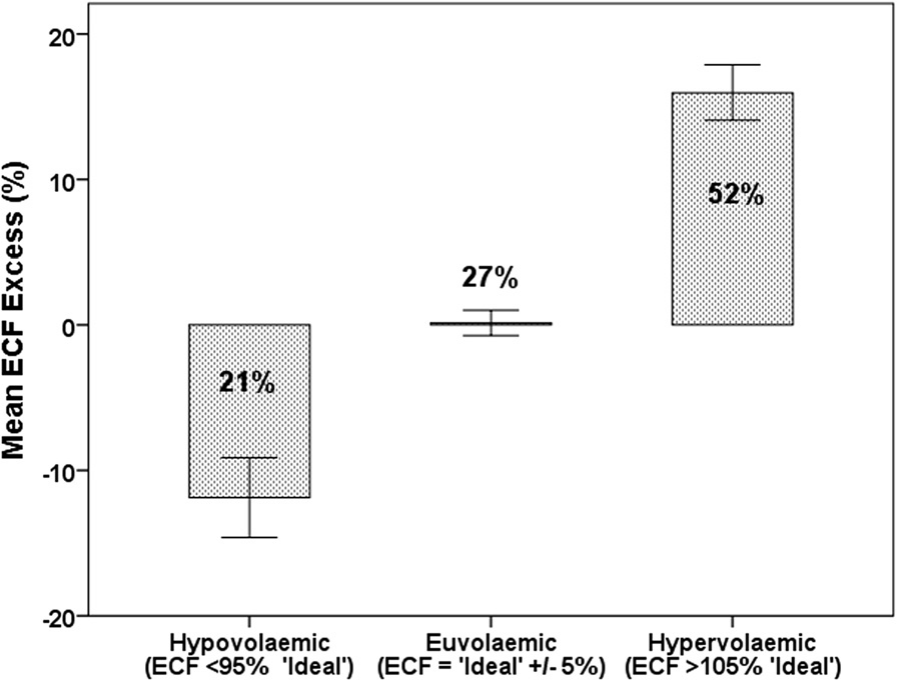
Mean % ECF excess based on bioimpedance estimates. Euvolaemic = ideal ECF [Chamney (25) +/− 5 %. Hypovolaemic = ECF < 95 % ideal. Hypervolaemic = ECF > 105 % ideal. Numbers in these categories indicate the percentage of the study population with ECF values in that category

Mean measured extracellular fluid volume (ECFV) pre-dialysis was 17.91 ± 3.98 l. Applying the Chamney equation the mean “ideal” ECFV was 17.09 ± 4.03 l (Table 1). Hence the mean excess ECFV (ECFV_excess_) was 0.82 ± 2.46 l. On this basis 45 patients (27 %) were within 5 % of their “ideal ECFV”(Euvolaemic), 36 patients (21 %) were more than 5 % below their “ideal ECF”(Hypovolaemic) and 89 patients (52 %) more than 5 % above it (Hypervolaemic) (Fig. 1).

### Natriuretic peptide levels

The median ANP levels were pre-dialysis 169 (IQR 208), and post-dialysis 79 (IQR 106) pg/ml. The median BNP level was 325 (IQR 1206) pg/ml (Table 1). Both pre- and post-dialysis ANP levels correlated significantly with BNP levels (rho = 0.760 and 0.688 respectively; p < 0.001 in both cases).

### Interrelationships of markers of volume status

#### Physical signs

##### Oedema

Patients with oedema were older and heavier than those without (Table 2). They also had higher systolic pressures pre-dialysis, lower serum albumin levels and a higher ECFV. There were no other significant differences, in particular no differences in natriuretic peptide levels.

**Table 2:** Comparison of Demographic, Clinical, Biochemical and Bioimpedance parameters in patients with oedema, raised JVP > 3 cm, and lung crepitations

	Oedema		p-value	JVP > 3 cm		p-value	Crepitations		p-value
	Yes	No		Yes	No		Yes	No	
Number	62	108		65	105		92	78	
Age (years)	69 ± 12	64 ± 14	0.021	66 ± 12	67 ± 13	NS	66 ± 13	66 ± 13	NS
Gender (%male)	68	68	NS	74	64	NS	74	60	0.058
Ethnicity (%white)	89	82	NS	88	82	NS	87	80	NS
Pre-dialysis weight (kg)	77.9 ± 18.2	71.2 ± 13.1	0.020	75.3 ± 15.5	72.6 ± 15.4	NS	77.0 ± 16.5	69.8 ± 13.1	0.002
KRU (ml/min)	0.38 (1.33)	0.26 (1.64)	NS	0.01 (1.06)	0.35 (1.82)	0.07	0.10 (1.22)	0.30 (1.83)	NS
Cardiac History (%)	50	43	NS	46	45	NS	47	44	NS
Charlson Comorbidity Index	6.1 ± 1.8	5.5 ± 2.3	0.053	5.4 ± 2.0	5.9 ± 2.2	NS	5.7 ± 2.1	5.7 ± 2.1	NS
Kt/V	1.29 ± 0.18	1.29 ± 0.23	NS	1.27 ± 0.16	1.30 ± 0.24	NS	1.26 ± 0.17	1.32 ± 0.24	0.066
Serum sodium (mmole/l)	137.1 ± 3.4	136.5 ± 3.2	NS	137.2 ± 3.0	136.7 ± 3.3	NS	136.9 ± 3.0	136.9 ± 3.5	NS
Albumin (g/l)	34.6 ± 4.8	36.1 ± 4.4	0.043	35.7 ± 4.3	35.3 ± 4.7	NS	36.0 ± 3.9	35.0 ± 5.3	NS
High CRP(>5 mg/l)	55	44	NS	54	44	NS	52	45	NS
Ultrafiltration Volume (l)	1.84 ± 1.10	1.63 ± 1.03	NS	2.05 ± 1.12	1.50 ± 1.00	0.001	1.97 ± 1.03	1.40 ± 1.01	<0.001
Systolic-pre (mmHg)	157 ± 25	148 ± 25	0.018	161 ± 26	145 ± 22	<0.001	158 ± 25	144 ± 23	<0.001
Diastolic-pre (mmHg)	80 ± 15	79 ± 15	NS	84 ± 16	76 ± 13	0.001	82 ± 15	76 ± 14	0.008
Systolic-post (mmHg)	140 ± 26	135 ± 26	NS	140 ± 29	134 ± 23	NS	140 ± 27	133 ± 24	NS
Diastolic-post (mmHg)	73 ± 13	74 ± 13	NS	75 ± 13	72 ± 12	NS	75 ± 13	71 ± 12	NS
Antihypertensive drugs (%)	76	72	NS	80	70	NS	75	72	NS
ECFV (l)	19.0 ± 4.5	17.3 ± 3.5	0.014	19.1 ± 3.9	17.1 ± 3.8	0.001	19.1 ± 4.1	16.5 ± 3.3	<0.001
ECFV Excess (l)	0.87 ± 3.28	0.79 ± 1.84	NS	1.57 ± 2.42	0.36 ± 2.38	0.002	1.12 ± 2.38	0.47 ± 2.52	0.085
ECFV Excess (% Ideal ECFV)	6.68 ± 16.72	5.44 ± 11.69	NS	10.08 ± 13.49	3.29 ± 13.24	0.002	7.37 ± 13.3	4.14 ± 14.03	NS
Excess ECFV (>5 %)	57	49	NS	68	42	0.002	58	46	NS
ANP-pre (pmol/l)	185 (268)	160 (199)	NS	201 (276)	149 (192)	0.023	197 (246)	147 (200)	0.042
ANP-post (pmol/l)	86 (123)	73 (99)	NS	101 (177)	72 (83)	0.038	88 (109)	70 (88)	NS
BNP (pmol/l)	401 (1157)	286 (1245)	NS	592 (1553)	281 (677)	0.014	410 (1369)	286 (675)	NS

*KRU* urea clearance, *CRP* serum C-Reactive Protein, *ECFV* extracellular fluid volume, *ANP* Atrial Natriuretic Peptide, *BNP* B-type Natriuretic peptide

##### Elevated Jugular Venous Pressure (JVP)

Those with elevated JVP levels > 3 cm tended to have less residual renal function than those without (Table 2). They also had higher pre-dialysis blood pressure levels – both systolic and diastolic, higher ECFV levels, and a higher proportion had a significantly expanded ECFV (Table 2). Levels of all natriuretic peptides were significantly higher in those with elevated JVP of this degree than in those without this sign.

##### Lung crepitations

Patients with lung crepitations were heavier than those without and also had higher ECFV levels, though minimal evidence of ECFV expansion. Blood pressure was higher especially pre-dialysis. Pre-dialysis ANP levels were also higher, though neither post-dialysis ANP nor BNP levels were significantly different between those with and those without this sign (Table 2).

The most powerful predictor of expanded ECFV was elevated JVP >3 cm. In fact of the 65 patients assessed as having this sign, 59 (91 %) also had lung crepitations or oedema or both.

#### Bioimpedance measures – excess ECF

The associations with bioimpedance-derived assessments of volume status – hypovolaemia, euvolaemia, and hypervolaemia – as defined above are shown in Table 3. The majority of patients (52 %) were identified as hypervolaemic, having an ECFV > 5 % above the ideal. These patients tended to be older, less heavy and of white ethnicity. Residual kidney function was significantly lower than in other groups. More had elevated CRP levels than euvolaemics. Clinical signs of hypervolaemia were also more pronounced. In particular post-dialysis systolic pressure was higher, and a significantly higher proportion had a raised JVP > 3 cm. UF rates were also higher. In addition natriuretic peptide levels were generally higher than in both other groups, especially BNP levels.

**Table 3:** Comparison of characteristic of hypovolaemic, euvolaemic and hypervolaemic groups

	Hypovolaemic <95 % ideal ECF	Euvolaemic Ideal ECF ± 5 %	Hypervolaemic >105 % ideal ECF	p-value
Number	36	45	89	
Demographic, clinical & laboratory factors				
Age (years)	63 ± 12	64 ± 14	^c^68 ± 13	0.085
% Male	56	71	71	NS
% White	^d^67	82	^c^92	0.002
Weight (kg)	^d^82 ± 20	75 ± 13	^a,c^70 ± 13	<0.001
Dialysis Vintage (months)	30 (34)	44 (46)	44 (53)	0.079
Diabetes (%)	39	22	24	NS
Cardiac History (%)	50	49	42	NS
Charlson Comorbidity Index	5.5 ± 2.3	5.5 ± 2.2	5.9 ± 2.0	NS
Haemoglobin (g/l)	116 ± 11	121 ± 13	^a,c^114 (15)	0.023
Serum albumin (g/l)	35.4 ± 5.4	36.5 ± 3.5	35.1 ± 4.7	NS
CRP > 5 mg/l	^b^58	31	^a^52	0.029
Serum sodium <136 mmol/l (%)	^d^47	29	27	0.079
KRU (ml/min)	^d^0.80 (2.66)	0.34 (1.77)	^c^0.04 (0.93)	0.011
Kt/V	1.32 ± 0.24	1.32 ± 0.25	1.29 ± 0.21	NS
Clinical assessment of volume status				
Post-dialysis systolic BP (mmHg)	^d^127 ± 20	135 ± 26	^c^141 ± 27	0.016
Lung Crepitations (%)	42	53	60	NS
Elevated JVP (>3 cm) (%)	^d^19	29	^a,c^51	0.002
Oedema (%)	42	24	41	NS
Natriuretic peptide levels				
ANP (pre-dialysis) (pmole/l)	147 (138)	149 (211)	^c^195 (274)	0.040
ANP (pre-dialysis) < 150 mmole/l (%)	53	53	^a,c^35	0.056
ANP (post-dialysis) (pmole/l)	62 (65)	62 (101)	^c^97 (116)	0.079
ANP (post-dialysis) < 100 pmole/l (%)	78	64	^c^51	0.015
BNP (pmole/l)	^d^228 (564)	268 (681)	^a,c^495 (1349)	0.003
BNP < 100 pmole/l (%)	25	33	^a,c^6	<0.001
Ultrafiltration characteristics				
Ultrafiltration volume (l)	1.35 (1.50)	1.70 (1.20)	1.80 (1.55)	NS
Ultrafiltration rate (ml/kg/h)	^d^6.2 ± 4.2	7.6 ± 4.3	^c^8.5 ± 5.1	0.046

The p-value refers to the significance of difference across the three groups by one-way ANOVA or the Kruskal Wallis test or Chi-square test as appropriate. ^a^ refers to a significant difference between hypervolaemic and euvolaemic groups. ^b^ refers to significance difference between hypovolaemic and euvolaemic groups. ^c^ refers to a significant difference between hypervolaemic group and the other two groups combined, ^d^ refers to a significant difference between hypovolaemic group and the other two groups combined

A small but significant minority (21 %) were hypovolaemic by the definition used (ECFV < 95 % of ideal). More of these patients were Non-white. They were heavier and had more residual kidney function than other groups. Again, more had elevated CRP levels than euvolaemics and a higher proportion had a serum sodium level < 136 mmol/l. Post-dialysis systolic pressure was lower than in other groups and fewer had a raised JVP > 3 cm. Ultrafiltration rates were lower. Generally natriuretic peptide levels were lower than in the hypervolaemic group, but similar to euvolaemic levels, though median BNP levels were slightly lower than in euvolaemics.

**Fig. 2:**
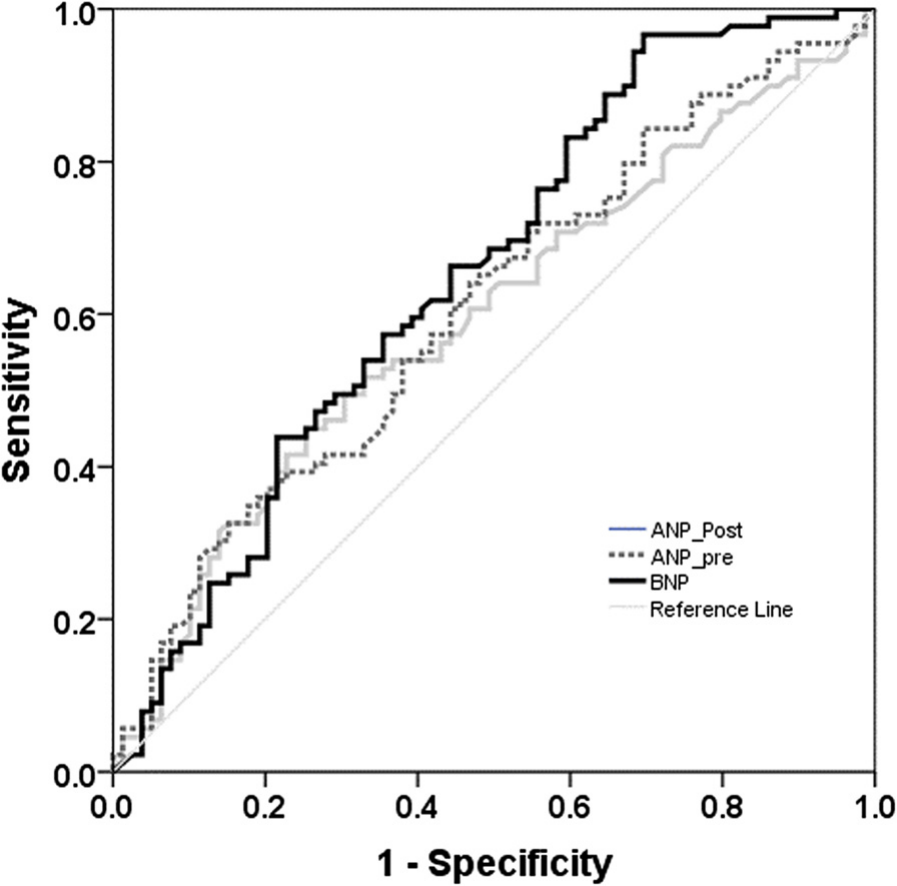
ROC curve indicating the utility of plasma levels of natriuretic peptides in predicting hypervolaemia as indicated by a bioimpedance-determined ECFV > 5 % above ideal ECF. For interpretation – see text

The utility of plasma levels of natriuretic peptides in predicting hypervolaemia as indicated by a bioimpedance-determined ECFV > 5 % above the ideal is demonstrated in the ROC curve in Fig. 2. Though BNP levels (AUC: 0.650: p = 0.001) were better predictors of hypervolaemia than pre- (AUC: 0.607: p = 0.017) or post-dialysis ANP levels (AUC: 0.595: p = 0.035), a cutoff level of 100 pmoles/l provided good sensitivity (94 %) but poor specificity (30 %). The utility of BNP in predicting hypovolaemia (ROC curve not shown) was even poorer with AUC for BNP of 0.616: p =0.037).

Regression models of determinants of hypervolaemia (ECFV > 105 % of ideal as determined by bioimpedance) are shown in Table 4. The baseline regression model included terms for gender, ethnicity, residual kidney function and pre-dialysis weight. The only clinical parameters which improved this model were post-dialysis blood pressure and elevated JVP > 3 cm. The only natriuretic peptide parameters which improved the model related to BNP, the most significant of which was serum BNP >100 pmol/l. Nagelkerke R square of the final model (Model C) was 0.435.

**Table 4:** Models of the determinants of hypervolaemia (ECFV >105 % of ideal as determined by bioimpedance)

	B	S.E.	Wald	p-value	Exp(B)
Model A: Baseline model (Nagelkerke R-square 0.272)					
Female v Male	−1.296	.476	7.409	.006	.274
Non-white v White	−1.859	.603	9.495	.002	.156
Residual Urea Clearance >1 ml/min	.678	.403	2.824	.093	1.969
Pre-dialysis weight (kg)	-.062	.016	14.648	.000	.939
Age and Charlson Comorbidity index - not significant in Model A					
Model B: Baseline model plus clinical factors (Nagelkerke R square 0.372)					
Post-dialysis systolic > 160 mmHg	1.190	.585	4.143	.042	3.288
Elevated JVP >3 cm	1.377	.422	10.656	.001	3.964
Parameters relating to pre-dialysis BP, oedema and basal lung crepitations – not significant in model B					
Model C: Baseline model plus clinical factors and natriuretic factors (Nagelkerke R square 0.435)					
BNP > 100 pg/ml	1.851	.602	9.468	.002	6.366
Parameters related to pre- and post-dialysis ANP not significant in model C					
Constant	2.675	1.420	3.547	.060	14.505

### Post-dialysis fluid balance

**Fig. 3:**
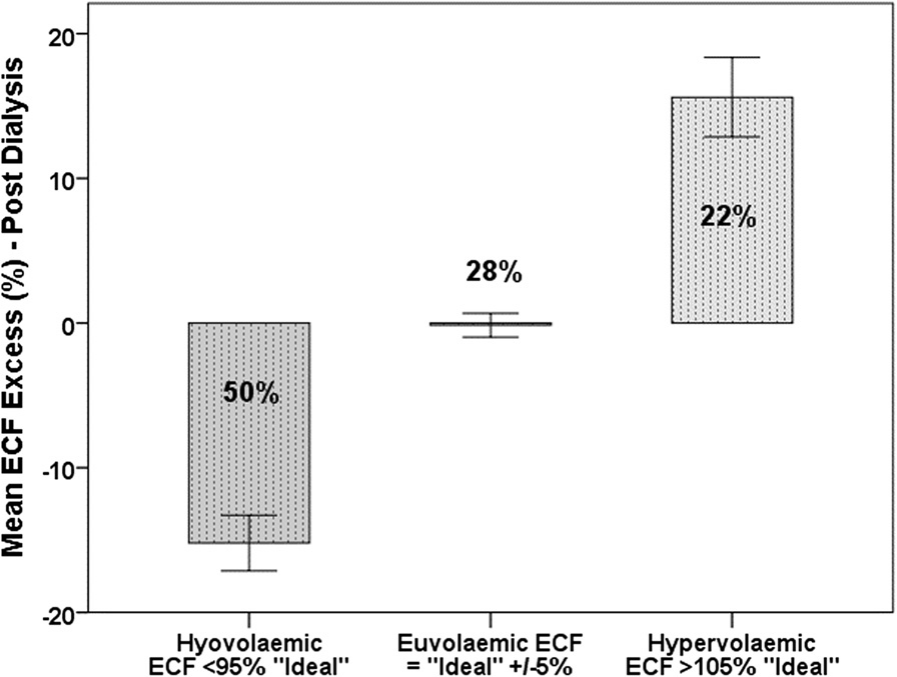
ROC curve depicting the association between natriuretic peptide levels and survival. For interpretation see text

Assuming that the UF volume removed during dialysis results in an equivalent reduction of the ECFV, the Excess ECF – post-dialysis can be calculated by subtracting the UFV volume from the Excess ECF – pre-dialysis. The results of this are shown in Fig. 3. The Excess ECF is expressed as a percentage of the ideal ECF in relation to the volume groups as defined above. It can be seen that post-dialysis the euvolaemic group is unchanged in size at 28 %, and that the size of the hypervolaemic group has reduced to 22 % at the expense of a major increase in the size of the hypovolaemic group which has risen to 50 %. This almost represents a mirror image of the pre-dialysis situation.

### Fluid overload and survival

**Fig. 4:**
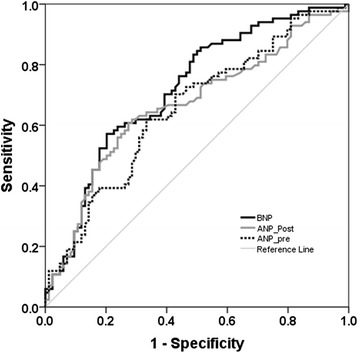
Mean % ECF excess post dialysis based on bioimpedance estimates modified by UF volume. Euvolaemic = ideal ECF [Chamney (25) +/− 5 %. Hypovolaemic = ECF < 95 % ideal. Hypervolaemic = ECF > 105 % ideal. Numbers in these categories indicate the percentage of the study population with ECF values in that category

Eighty-four patients (49 %) died, 32 (19 %) were transplanted and one (0.6 %) transferred out during the 5 year follow-up. The association of natriuretic peptides and survival was explored in a series of ROC curves (Fig. 4). BNP (AUC: 0.719: p < 0.001) provided a better prediction of 5 year mortality than pre- (AUC: 0.652: p = 0.001) or post-dialysis ANP levels (AUC: 0.665: p <0.001). For BNP a cut-off level of 100 pmole/l provided a high sensitivity (93 %) but poor specificity (30 %). A series of Cox Regression models were constructed (Table 5). The baseline model included terms for age, sex, ethnicity,, predialysis weight, residual kidney function (KRU > 1 ml/min), CCI, Kt/V, serum albumin and raised CRP (>5 mg/l). In separate models (Models 1, 2 and 3) terms for log transformed pre-dialysis ANP, post-dialysis ANP and BNP were added to the baseline model. In these models pre-dialysis and post-dialysis ANP terms were of only marginal significance, unlike that for BNP the term for which was highly significant. Furthermore neither log pre-dialysis ANP nor log post-dialysis ANP was significant when added to Model 3 whilst log BNP retained its significance.

**Table 5:** Effect of ANP and BNP on survival in Cox Regression models of survival in 170 Haemodialysis patients

		B	SE	Wald	p-value	Exp(B)
Model 1	Baseline + Log pre-dialysis ANP	.204	.136	2.263	.132	1.227
Model 2	Baseline + Log post-dialysis ANP	.235	.147	2.560	.110	1.265
Model 3	Baseline + Log BNP	.261	.093	7.916	.005	1.299

The baseline model included age, sex, ethnicity, pre-dialysis weight, residual urea clearance > 1 ml/min, Charlson Comorbidity Index, serum albumin, and elevated CRP (>5 mg/l). Log BNP was a significant determinant of survival in this model unlike Log pre-dialysis ANP and Log post-dialysis ANP

## Discussion

Fluid overload is common in HD patients and is associated with significant morbidity including excess risk of cardiovascular disease as well as mortality. Whilst clinical assessment remains the cornerstone, bioimpedance methods are increasingly used to supplement this. Several studies have utilised bioimpedance to provide objective assessment of volume status in HD patients [1, 26–29] This study explored the potential for estimates of blood levels of natriuretic peptide to provide additional clinically useful information. It also examines their role in predicting survival.

Bioimpedance analysis in this study revealed that though the majority of patients (52 %) had excess ECF, a significant proportion (21 %) were hypovolaemic. Clinical signs associated with fluid overload were prevalent in this group of patients – oedema (37 %), raised JVP > 3 cm (38 %) and basal crepitations (54 %). Elevated JVP >3 cms was the most powerful clinical predictor of expanded ECF volume, and the only independent clinical predictor of expanded ECF volume in multivariate analysis. It is also pertinent that 91 % of the patients who were found to have a raised JVP of this degree also had lung crepitations or oedema or both.

Patients in the study had high levels of pre dialysis ANP, post dialysis ANP as well as BNP. There were no significant differences in the level of pre dialysis ANP and BNP with regards to gender but men in the study tended to have higher levels of post dialysis ANP. A number of studies have reported that natriuretic peptide levels are elevated in HD patients and it has been suggested that factors such as fluid overload, left ventricular dysfunction and impaired renal clearance contribute to this [19, 22, 30–33]. Pre and post dialysis ANP levels correlated strongly with each other as well as with BNP which is unsurprising.

We found that BNP was a stronger predictor of fluid overload than pre and post dialysis ANP. This was evident both in terms of its relationship with clinical markers of volume status, particularly elevated JVP (Table 2) and in terms of its relationship with Excess ECFV (Tables 2 and 3). This is perhaps surprising as ANP is released predominantly by atria and has much shorter half-life compared to BNP, which is predominantly released by the ventricle. This finding suggests that cardiac function may be a major confounder for fluid overload leading to excess ECF. It has been suggested that ANP is more responsive to intravascular volume changes in HD patients than BNP whilst BNP may be more reflective of cardiac dysfunction [34]. Our findings also raise the possibility of a dissociation between these biomarkers in relation to fluid overload – with changes in ANP being more reflective of acute volume change and BNP more reflective of a chronically fluid overloaded state.

Bioimpedance analysis was used to classify patients in the study to three groups based on their excess ECF, both pre and post dialysis. Patients who were classified as being fluid overloaded pre dialysis had significantly higher levels of natriuretic peptides compared to euvolaemic and hypovolaemic subjects. These patients had lower level of residual renal function compared to remainder of the patients. In addition, their post dialysis systolic blood pressure remained high despite the high ultrafiltration rate. A significantly higher proportion of patients (55 %) were classified as hypovolaemic post dialysis, mirroring the proportion of hypervolaemic group pre dialysis. Likewise, patients who were hypovolaemic tended to have lower post dialysis systolic blood pressure and lower ultrafiltration rate. Intra dialytic hypotension, a marker of hypovolaemia, has been increasingly recognised as a marker of adverse outcomes in HD patients. In addition to asymptomatic myocardial ischaemia [35], it is also associated with other adverse effects such as accelerated decline in residual renal function [36] and increased risk of vascular access thrombosis [37].

Patients on renal replacement therapy have high risk of cardiovascular disease and fluid overload is a major confounder for cardiac disease including left ventricular dysfunction. In ROC analysis we found that BNP was a stronger predictor of 5-year survival in this cohort of patients compared to pre- and post-dialysis ANP. We also found that BNP but not ANP was an independent predictor of mortality in Cox regression analysis in this group of patients. Several studies have shown the relationship between BNP and survival in HD patients and some have utilised NT proBNP rather than BNP [3, 38–41]. BNP and NT proBNP were compared in an earlier study and there were only marginal differences between them with regards to survival in HD patients [31]. None to our knowledge has compared the contributions to survival prediction of ANP and BNP in this setting.

## Conclusions

In conclusion bioimpedance analysis suggested that ECFV expansion was present in over 50 % of patients pre-dialysis though a significant proportion (21 %) had a depleted ECFV in this setting. The situation was reversed post-dialysis. A raised JVP >3 cm was the most reliable clinical sign of ECFV expansion inferred from bioimpedance measurements and natriuretic peptide levels. The vast majority of patients with this sign also had lung crepitations or peripheral oedema or both. We found BNP to be a stronger predictor of volume overload in this setting than pre- or post-dialysis ANP. BNP was also a stronger predictor of survival over an extended follow up period of 5 years. We found no clear role for measurement of ANP levels in this setting, though changes in ANP may be a strong indicator of acute changes in volume status. Further work is necessary to explore the possible role of monitoring these levels of peptides in the management of volume status and cardiovascular risk in HD patients.

## Abbreviations

ANOVA: Analysis of Variance
ANP: Atrial Natriuretic Peptide
AUC: Area under curve
BNP: B-type Natriuretic Peptide
BP: Blood Pressure
CCI: Charlson Comorbidity Index
CRP: C Reactive Protein
ECF: Extracellular Fluid
ECFV: Extracellular Fluid Volume
EDTA: Ethylenediaminetetraacetic Acid
HD: Haemodialysis
IQR: Intra-quartile Range
JVP: Jugular Venous Pressure
KRU: Renal Urea Clearance
Kt/V: Urea Clearance normalised to total body water
ROC: Receiver Operator Characteristic
RRT: Renal Replacement Therapy
SPSS: Statistical Package for Social Services
Td: Duration of haemodialysis session
UFR: Ultrafiltration Rate
UFV: Ultrafiltration Volume
